# Patterns and characteristics of nicotine pouch use among adults with a history of cigarette smoking

**DOI:** 10.3389/fpubh.2026.1806892

**Published:** 2026-06-15

**Authors:** Hajed M. Al-Otaibi, Malik A. Althobiani

**Affiliations:** 1 Department of Respiratory Therapy, Faculty of Medical Rehabilitation Sciences, King Abdulaziz University, Jeddah, Saudi Arabia; 2 Respiratory Therapy Unit, King Abdulaziz University Hospital, King Abdulaziz University, Jeddah, Saudi Arabia; 3 UCL Respiratory, University College London, London, United Kingdom

**Keywords:** craving, nicotine dependence, nicotine pouches, smoking, tobacco

## Abstract

**Background:**

Nicotine pouches (NP) are emerging as a potentially safer alternative to tobacco smoking. However, data on NP users with a history of tobacco smoking are limited. We assessed the patterns of NP use, user characteristics, and perceptions among adults with a history of cigarette smoking.

**Methods:**

A cross-sectional survey was conducted using a self-administered questionnaire to enroll adults with a history of regular cigarette smoking. Data on sociodemographics, smoking history, NP use patterns, and NP-related urges and perceptions were collected. Group differences were analyzed using χ^2^ tests, and multivariable logistic regression identified factors associated with high-intensity NP use.

**Results:**

A total of 711 adults with a history of combustible cigarette smoking were included, of whom 693 were analyzed. Among them, 560 were current users of NP who had ceased cigarette smoking (NP users) and 133 were current cigarette smokers who had not adopted NP (non-users). We found no difference between the two groups in gender, educational status, income, marital status, years of smoking, and time to first cigarette (all *p* > 0.05). A total of 236 NP users (42.1%) reported high-intensity NP use (≥10 pouches/day) and a total of 363 NP users (64.8%) used their first NP within 30 min of waking. About half of the NP users reported reduced craving intensity (*n* = 312; 55.7%), and experienced milder withdrawal symptoms (*n* = 245; 43.8%) compared with smoking. A total of 339 (60%) rated NP as extremely or very effective for smoking cessation. Higher cigarette per day (CPD) was associated with increased odds of high-intensity NP use (≥10 pouches/day): 21–30 CPD (OR = 2.70; 95% CI: 1.62–4.50) and >30 CPD (OR = 3.15; 95% CI: 1.40–7.10).

**Conclusion:**

The present findings suggest that adults with a history of cigarette smoking report NP use patterns that appear broadly similar to their prior smoking behaviors. Users reported that cravings and withdrawal symptoms during NP use were similar to or lower than those recalled during smoking. Although most users viewed NP as effective for cessation, it may instead serve as an alternative nicotine source rather than a cessation tool.

## Introduction

Tobacco use remains a major public health concern in Saudi Arabia, with a tobacco use prevalence of 19% among adults (30% among males) (1). The nicotine pouches (NP) market has grown rapidly in the Kingdom, with locally manufactured products becoming widely available. Recent cross-sectional surveys report NP awareness rates of 59–70% among Saudi adults, with ever-use prevalence of 11–21% (2–4). Understanding NP use patterns in this population is important as the regulatory framework for NP in Saudi Arabia is still evolving.

NP are emerging as a new category of tobacco-free nicotine products (5). These products consist of a small cellulose-based matrix infused with nicotine. The median pH of NP was reported to be 8.8 (6). An increase in pH (alkalinity) raises the proportion of unionized nicotine, which may enhance its absorption through the oral mucosa. Therefore, users place the pouch between the gum and the lip to allow sustained nicotine delivery through the oral mucosa. NP are formulated to deliver varying amounts of nicotine depending on product strength and other contributing factors. In analyzed commercial nicotine-pouch products, total nicotine content has been reported to range from 0.89 to 6.73 mg/pouch (7). A US sales analysis reported that 6 mg, 4 mg, and 3 mg nicotine concentration levels were the most commonly sold during the study period (8). The global market for NP has expanded substantially in recent years. In the United States, sales of NP increased over time, particularly from mid-2021 onward (8, 9). At present, an increasing number of manufacturers worldwide are introducing NP to the market (10).

There is evidence that NP are highly appealing to current tobacco users, especially those who are active smokers, use smokeless tobacco, and electronic cigarette users (11, 10). In Saudi Arabia, current cigarette smokers were approximately 9.5 times more likely to have used NP than non-smokers (2). This may be a result of how these products are being promoted. Manufacturers of NP often present them as a potentially safer alternative to both combusted and non-combusted tobacco products. This may convey indirect messages that could encourage conventional tobacco users to consider switching to NP (5). These strategies may contribute to capturing a meaningful share of the tobacco use market. Through marketing phrases such as “tobacco-free” and “smoke-free,” manufacturers aim to dissociate these products from the traditional mental image of tobacco use (12). This perception has likely encouraged some researchers to investigate the effectiveness of NP as a smoking cessation tool (13–15). However, despite the limitations of these studies, the evidence for their effectiveness as a smoking cessation intervention remains limited and inconclusive.

Currently, there is a limited number of large-scale studies examining the prevalence of NP use among adults who currently smoke or who have a history of cigarette smoking. One of these studies indicates that smokers and adults with a history of cigarette smoking are very likely to try NP (16). NP may contain fewer toxicants than combusted tobacco products; however, this does not imply they are risk-free, and their long-term safety profile remains unknown (17). Their potential short- and long-term health risks still need to be identified. However, tobacco users who feel ambivalent about conventional tobacco and its health risks may find NP appealing and more attractive. Self-reported experiences such as craving relief and fewer respiratory symptoms typically associated with smoking, including cough and increased sputum, may contribute to the appeal of NP use. Therefore, some users perceive NP as a potential smoking cessation aid (11). Regardless of the intended purpose, nicotine is an addictive substance.

This study aims to describe NP adoption and use patterns among adults with a history of combustible cigarette smoking and to explore associations with self-reported craving, withdrawal, and perceptions regarding cessation. It also aims to compare prior smoking behaviors with current NP use to identify factors underlying NP adoption.

## Materials and methods

### Study design

This study employs a cross-sectional design using a self-administered survey to examine the characteristics of adults with a history of combustible cigarette smoking, categorized as NP users (current and regular NP users) and non-users (current cigarette smokers not using NP). The survey also aims to assess their usage patterns and perceptions regarding NP. The survey gathers data on participants’ sociodemographic characteristics, smoking history, cigarette smoking behavior assessed using the Fagerström Test for Nicotine Dependence (FTND) (18), current patterns of NP use evaluated with the modified Fagerström Test for Nicotine Dependence -Smokeless Tobacco (FTND-ST) (19), and perceptions of NP as a potential smoking cessation aid.

### Study population and sampling

Participants were adults (≥18 years) residing in Saudi Arabia with a history of regular combustible cigarette smoking, defined as having smoked at least 100 cigarettes in their lifetime and having smoked daily or on most days during their period of regular smoking. Individuals who were non-smokers, users of other tobacco products (e.g., shisha/hookah), smokeless tobacco users, electronic cigarette users, or those aged <18 years were excluded. NP Users were defined as participants who reported current NP use and who had ceased combustible cigarette smoking prior to or upon adopting NP (i.e., former smokers who completely switched to NP)—no frequency of NP use was determined. Current cigarette smoking status was assessed via a survey routing item; only participants who reported having ceased cigarette smoking were directed to the NP use section of the questionnaire. This item was implemented as skip logic within the survey platform (SurveyMonkey) and was not exported as a data variable. Non-Users were defined as participants who reported continued current cigarette smoking and no current NP use.

Segmenting nicotine product users into distinct exposure categories is considered best practice for maximizing analytical precision in observational studies of tobacco and nicotine products (20). Conceptually, a combustible cigarette smoker who initiates NP use may transition into one of four states: (I) trial without adoption, in which the individual reverts to exclusive cigarette use; (II) concurrent dual use of cigarettes and NP; (III) complete switching from exclusive cigarette use to exclusive NP use; or (IV) cessation of all nicotine products (20). The present study captured States I and III; dual users (State II) were excluded by the survey design, and individuals who had achieved complete nicotine cessation (State IV) would not be expected to participate in a survey of current NP users. Accordingly, NP Users represent complete switchers (State III) and Non-Users represent current smokers who did not adopt NP (State I).

Recruitment used a non-probability sampling strategy, convenience sampling, to target current or former cigarette smokers who currently use NP. The survey was distributed through social media platforms, including WhatsApp, Twitter (X), Facebook, and Telegram, between July and December 2025. The survey was administered in Arabic, the primary language of the target population. The recruitment materials specifically targeted individuals with prior NP experience who had a history of cigarette smoking. Non-Users therefore represent individuals who had been exposed to NP but reported continued current cigarette smoking and no current NP use at the time of survey completion (i.e., individuals who tried NP but did not adopt them).

### Sample size

The sample size was calculated using the standard formula commonly applied for cross-sectional surveys. The large population prevalence formula was used to estimate the required sample size: n = Z^2^ × p (1 − p)/e^2^. Assuming *Z* = 1.96 (95% confidence), *p* = 0.50, and *e* = 0.05, the minimum sample size was calculated as 385 participants. An additional 30% was added to the estimated required sample size to account for duplicate responses, incomplete items, and potential non-response. Therefore, the final target minimum sample size was estimated not to be less than 500 participants.

### Questionnaire development and measures

The survey instrument was developed using existing literature and validated measures where available. It comprises the following domains:

#### Sociodemographics

Key sociodemographic characteristics of participants, including age, gender, educational level, and monthly income, were collected.

#### Tobacco history

Data were collected on duration of combustible cigarette use, and all items of the FTND to assess the level of tobacco dependence during participants’ period of regular cigarette smoking (State A: period of exclusive cigarette use, before NP initiation). Participants were instructed to complete the FTND based on their period of most regular cigarette smoking. This retrospective approach is subject to recall bias, particularly among those who may have ceased smoking some time ago. Data on NP use were collected, including duration of use, nicotine strength (mg per pouch, as labeled by the manufacturer), usage patterns, preferred flavors, and all items of the modified FTND-ST to evaluate NP use patterns. The modified FTND-ST was not originally designed or validated to assess dependence on NP. Although some studies have used it for this purpose (21), its use in NP users should be interpreted cautiously. The FTND-ST includes six items, of which four were retained in their original form and two were adapted. The original item, “How often do you intentionally swallow tobacco juice?” was replaced with, “How many pouches do you use per day?” Similarly, the question, “Do you chew more frequently during the first hours after waking than during the rest of the day?” was modified to, “Do you use more than one pouch at a time to increase nicotine strength?” Since NP share some behavioral characteristics with smokeless tobacco products, the adapted instrument may capture patterns relevant to NP use. Items were scored following the original FTND-ST rubric where applicable: time to first pouch after waking (>60 min = 0, 31–60 = 1, 6–30 = 2, within 5 min = 3), difficulty refraining in restricted places (no = 0, yes = 1), which use period would be most hated to give up (any other = 0, the first one in the morning = 1), and use when ill (no = 0, yes = 1). For the two adapted items, pouches per day was scored as 0 (1–3), 1 (4–9), 2 (10–15), or 3 (>15), and use of multiple pouches at once was scored as 0 (never), 1 (rarely), 2 (sometimes), or 3 (regularly), yielding a total possible score range of 0–12. These items characterize current NP use patterns (State B: current nicotine use state; NP Users correspond to State B-III and Non-Users to State B-I). However, because two of six items were replaced with items measuring different constructs (consumption volume and dose-stacking rather than ingestion behavior and temporal use pattern), the adapted instrument should not be interpreted as a validated measure of nicotine dependence. Both replacement items were developed by the authors for this study and were not taken from Dowd et al. (21).

#### Urges and perceptions

Participants’ urges to use cigarettes and NP were assessed. Specifically, participants rated (A) craving intensity during current NP use relative to prior cigarette smoking and (B) withdrawal symptoms severity relative to prior cigarette smoking; participants who had not stopped using the product long enough to assess withdrawal symptoms could select “N/A.” In addition, their perceptions of NP as a potential smoking cessation tool were collected. These perceptions included perceived effectiveness of NP as a smoking-cessation aid and likelihood of recommending NP to other smokers.

### Instrument validity and pilot testing

The questionnaires were reviewed by two subject matter experts to assess content validity. They evaluated item clarity, relevance, cultural appropriateness, and comprehensibility of the questionnaire items. In addition, they assessed the questionnaire for grammatical and vocabulary issues that might affect understanding of the questions or lead to misinterpretation. Items that were unclear or had structural issues were revised and modified accordingly. The questionnaire was then finalized and sent to a third subject-matter expert to evaluate the clarity, simplicity and readability of all items. Minor adjustments were applied accordingly to finalize the questionnaire. Flavor categories were developed based on a review of options available on popular NP brands in Saudi Arabia, published literature (21, 22), and expert review during content validation.

### Data collection procedures

The survey was hosted on a secure online platform (SurveyMonkey). Informed consent was obtained electronically. The consent materials explained the voluntary nature of participation, confidentiality of responses, and by selecting “Yes,” participants provided their electronic consent. The estimated time to complete the survey was also provided. The full survey instrument, including all items, response options, and their sources, is provided as Supplementary file S1.

## Statistical analysis

Descriptive statistics were used to characterize the study population. Categorical variables, including age, sex, education, income, marital status, and smoking history, are presented as frequencies (n) and percentages (%). For descriptive proportions reported in Table 1, 95% confidence intervals (95% CIs) were calculated using the Wilson score method with the full NP-user sample (*N* = 560) to maintain consistency with the reported percentages. Differences in participant characteristics between NP users and non-users were assessed using Pearson’s chi-square tests (χ^2^), with Fisher’s exact test applied when expected cell counts were <5. All tests were two-tailed, and *p* < 0.05 was considered statistically significant.

**Table 1:** Baseline demographic and clinical characteristics of the study population (*N* = 693).

Variable	Category	Total (*N* = 693)	NP users (*n* = 560)	Non-users (*n* = 133)	*p*-value
Age group	18–25	131 (18.9%)	111 (19.8%)	20 (15.0%)	0.017
	26–35	277 (40.0%)	232 (41.4%)	45 (33.8%)	
	36–45	208 (30.0%)	165 (29.5%)	43 (32.3%)	
	46–55	65 (9.4%)	45 (8.0%)	20 (15.0%)	
	56–65	8 (1.2%)	4 (0.7%)	4 (3.0%)	
	>65	4 (0.6%)	3 (0.5%)	1 (0.8%)	
Gender	Female	26 (3.8%)	17 (3.0%)	9 (6.8%)	0.075
	Male	667 (96.2%)	543 (97.0%)	124 (93.2%)	
Education level	High school or less	104 (15.0%)	80 (14.3%)	24 (18.0%)	0.530
	Post-secondary diploma	151 (21.8%)	122 (21.8%)	29 (21.8%)	
	Bachelor’s degree	331 (47.8%)	274 (48.9%)	57 (42.9%)	
	Graduate degree	107 (15.4%)	84 (15.0%)	23 (17.3%)	
Monthly income	<5,000 SAR*	158 (22.8%)	124 (22.1%)	34 (25.6%)	0.709
	5,001–10,000 SAR	151 (21.8%)	126 (22.5%)	25 (18.8%)	
	10,001–15,000 SAR	173 (25.0%)	136 (24.3%)	37 (27.8%)	
	15,001–20,000 SAR	127 (18.3%)	105 (18.8%)	22 (16.5%)	
	>20,001 SAR	84 (12.1%)	69 (12.3%)	15 (11.3%)	
Marital status	Not married (single)	293 (42.3%)	240 (42.9%)	53 (39.8%)	0.594
	Married	400 (57.7%)	320 (57.1%)	80 (60.2%)	
Cigarette per day	10 or fewer	207 (29.9%)	168 (30.0%)	39 (29.3%)	0.717
	11–20	343 (49.5%)	280 (50.0%)	63 (47.4%)	
	21–30	108 (15.6%)	83 (14.8%)	25 (18.8%)	
	31 or more	35 (5.1%)	29 (5.2%)	6 (4.5%)	
Time to first cigarette	Within 5 min	186 (26.8%)	146 (26.1%)	40 (30.1%)	0.249
	6–30 min	243 (35.1%)	201 (35.9%)	42 (31.6%)	
	31–60 min	116 (16.7%)	99 (17.7%)	17 (12.8%)	
	After 60 min	148 (21.4%)	114 (20.4%)	34 (25.6%)	
Years of smoking	≤5 years	172 (24.8%)	141 (25.2%)	31 (23.3%)	0.387
	6–10 years	196 (28.3%)	156 (27.9%)	40 (30.1%)	
	11–15 years	151 (21.8%)	128 (22.9%)	23 (17.3%)	
	>15 years	174 (25.1%)	135 (24.1%)	39 (29.3%)	
FTND score	Mean ± SD	5.02 ± 2.40	5.04 ± 2.40	4.97 ± 2.42	0.699
FTND category	Very low (0–2)	119 (17.2%)	99 (17.7%)	20 (15.0%)	0.805
	Low (3–4)	143 (20.6%)	112 (20.0%)	31 (23.3%)	
	Moderate (5)	97 (14.0%)	76 (13.6%)	21 (15.8%)	
	High (6–7)	223 (32.2%)	183 (32.7%)	40 (30.1%)	
	Very high (8–10)	101 (14.6%)	82 (14.6%)	19 (14.3%)	
	Missing	11 (1.6%)	8 (1.4%)	3 (2.3%)	

*SAR, Saudi Riyals; FTND, Fagerström Test for Nicotine Dependence.

To identify predictors of high-intensity consumption (defined as ≥10 pouches/day), a multivariable logistic regression model was used. Variables were selected *a priori* based on theoretical relevance, previous literature on nicotine-use behaviors, and their known association with consumption intensity. Sex, age group, monthly income, years of smoking, cigarettes per day (CPD), and FTND score were included in the model. Where cell counts were insufficient for stable regression estimates, adjacent categories were collapsed. Specifically, age groups 56–65 (*n* = 4) and >65 (*n* = 3) were collapsed into a single ≥56 years category. All other variables were entered using the categories presented in Table 2. The regression was conducted using complete-case analysis; participants with missing values on any covariate were excluded. Of 560 NP Users, 8 had missing FTND scores and 1 had missing pouch consumption data, leaving 551 for the regression analysis. No imputation was performed given the low proportion of missing data (<2%). All recoding decisions are noted in the relevant tables. All analyses were conducted using Python (version 3.x, with pandas, scipy, and statsmodels libraries).

**Table 2:** Patterns of NP usage among NP users.

Characteristic	NP users (*n* = 560)
Duration of NP use	
<6 months	154 (27.5%) [24.0–31.3]
6 months to 1 year	153 (27.3%) [23.8–31.2]
1–2 years	204 (36.4%) [32.5–40.5]
2–3 years	37 (6.6%) [4.8–9.0]
>3 years	12 (2.1%) [1.2–3.7]
Pouches used per day	
1–3	60 (10.7%) [8.4–13.5]
4–9	262 (46.8%) [42.7–50.9]
10–15	188 (33.6%) [29.8–37.6]
>15	48 (8.6%) [6.5–11.2]
Missing	2 (0.4%)
Time to first pouch after waking	
Within 5 min	170 (30.4%) [26.7–34.3]
6–30 min	193 (34.5%) [30.6–38.5]
31–60 min	111 (19.8%) [16.7–23.3]
After >60 min	84 (15.0%) [12.3–18.2]
Missing	2 (0.4%)
Preferred nicotine strength	
3 mg	29 (5.2%) [3.6–7.3]
6 mg	83 (14.8%) [12.1–18.0]
10 mg	405 (72.3%) [68.5–75.9]
15 mg	18 (3.2%) [2.0–5.0]
20 mg	18 (3.2%) [2.0–5.0]
Missing	7 (1.3%)
Change in nicotine strength since starting	
Switch between strengths as needed	54 (9.6%) [7.5–12.4]
Started high, decreased gradually	74 (13.2%) [10.7–16.3]
Started low, increased gradually	94 (16.8%) [13.9–20.1]
Changed strength up and down multiple times	41 (7.3%) [5.4–9.8]
No change since starting	288 (51.4%) [47.3–55.5]
Missing	9 (1.6%)
Use multiple pouches at once	
No, never	168 (30.0%) [26.4–33.9]
Yes, rarely	197 (35.2%) [31.3–39.2]
Yes, sometimes	137 (24.5%) [21.1–28.2]
Yes, regularly	54 (9.6%) [7.5–12.4]
Missing	4 (0.7%)
Preferred pouch flavor	
Classic tobacco	25 (4.5%) [3.0–6.5]
Berries (strawberry, blueberry)	39 (7.0%) [5.1–9.4]
Tropical fruits (mango, pineapple)	5 (0.9%) [0.4–2.1]
Citrus fruits (lemon, orange)	9 (1.6%) [0.8–3.0]
Coffee/caffeine	9 (1.6%) [0.8–3.0]
Mint	374 (66.8%) [62.8–70.6]
Sweet flavors (vanilla, caramel)	3 (0.5%) [0.2–1.6]
Herbal/natural flavors	28 (5.0%) [3.5–7.1]
Unflavored	48 (8.6%) [6.5–11.2]
Missing	20 (3.6%)
Importance of flavor in choosing NP	
Not important at all	34 (6.1%) [4.4–8.4]
Slightly important	19 (3.4%) [2.2–5.2]
Moderately important	67 (12.0%) [9.5–14.9]
Important (but not the most important)	125 (22.3%) [19.1–26.0]
Very important (most important factor)	314 (56.1%) [51.9–60.1]
Missing	1 (0.2%)
Use NP when ill	
No	239 (42.7%) [38.6–46.8]
Yes	318 (56.8%) [52.7–60.8]
Missing	3 (0.5%)

Percentages and 95% confidence intervals were calculated using the Wilson score method with the full NP-user sample (*N* = 560) to maintain consistency with the descriptive values reported throughout the manuscript. Confidence intervals are shown for substantive response categories only; missing data are presented as n (%).

## Results

A total of 711 participants responded to the survey, and 18 participants (2.5%) were duplicates or excluded due to incomplete participant demographic or smoking history data. The final analysis included 693 adults with a history of combustible cigarette smoking, of whom 560 (80.8%) were grouped as NP Users (former smokers who had completely switched to NP) and 133 (19.2%) as Non-Users (current cigarette smokers). The participant demographic and clinical characteristics of the study population are shown in Table 1. NP Users were younger than Non-Users (*p* = 0.017), with the largest proportion in the 26–35 age group (41.4%), whereas Non-Users were seen more frequently in age groups above 46 years. Between-group comparisons were non-significant for gender (*p* = 0.075), education (*p* = 0.530), monthly income (*p* = 0.709), or marital status (*p* = 0.594). Baseline smoking behaviors, specifically daily cigarette volume, cumulative years of smoking, and time to first cigarette, showed no meaningful differences between groups (all *p* > 0.05).

**Figure 1:**
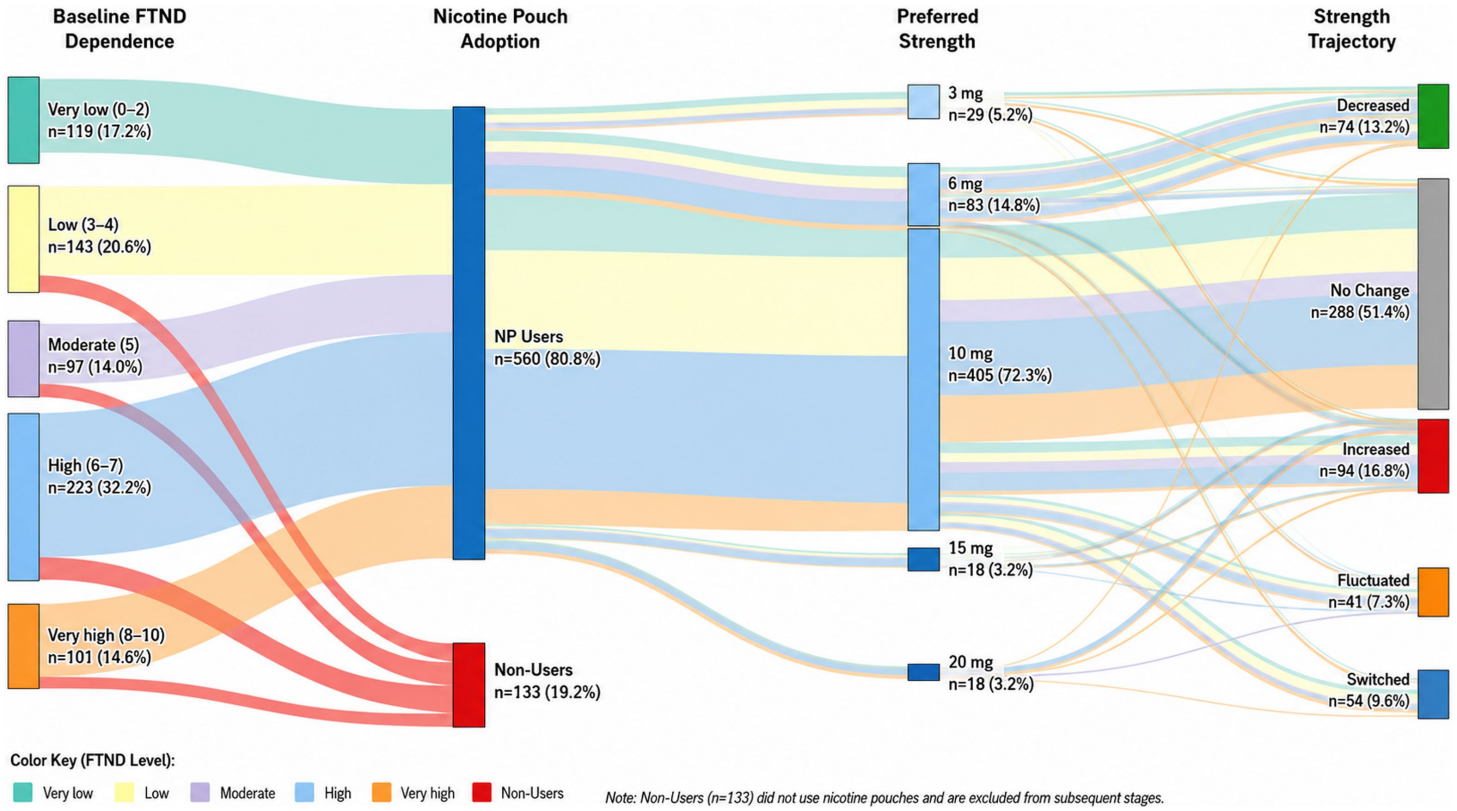
Trajectory of nicotine dependence, pouch adoption and strength patterns. Sankey diagram showing participant flow from baseline nicotine dependence (FTND) to nicotine pouch adoption, preferred pouch strength, and strength trajectory (*N* = 682; 11 participants with missing FTND data are not included). Baseline FTND categories were very low (0–2), low (3–4), moderate (5), high (6–7), and very high (8–10). Participants were grouped as NP users (*n* = 560, 80.8%) or non-users (*n* = 133, 19.2%); non-users were current smokers who had not adopted NP and did not contribute to later stages. Preferred strength options ranged from 3 to 20 mg, with 10 mg most common (405/560, 72.3%). Strength trajectory categories were decreased (74/560, 13.2%), no change (288/560, 51.4%), increased (94/560, 16.8%), fluctuated (41/560, 7.3%), and switching as needed (54/560, 9.6%). Colors correspond to FTND category.

Figure 1 presents the transition from nicotine dependence to NP adoption and follow-up strength choices among 693 participants. Baseline dependence was assessed with the FTND and was most frequent in the high range (6, 7) (223/693, 32.2%), followed in frequency by low (3, 4) (143/693, 20.6%), very low (0–2) (119/693, 17.2%), very high (8–10) (101/693, 14.6%), and moderate (5) (97/693, 14.0%); FTND data were missing for 11 participants (1.6%).

Among all participants, 560/693 (80.8%) had adopted NP, while 133/693 (19.2%) were current smokers who had not adopted NP and were therefore not represented in subsequent stages. Among NP users, 10 mg was the most frequently chosen strength (405/560, 72.3%), remaining participants chose 6 mg (83/560, 14.8%), 3 mg (29/560, 5.2%), 15 mg (18/560, 3.2%), or 20 mg (18/560, 3.2%); preferred strength was missing for 7 NP users (1.3%). When examining strength-change patterns, most NP users reported no change (288/560, 51.4%), followed in frequency by increased strength (94/560, 16.8%), decreased strength (74/560, 13.2%), switching as needed (54/560, 9.6%), and inconsistent strength pattern (41/560, 7.3%); trajectory data were missing for 9 NP users (1.6%). Flows are color-coded by FTND category.

### Patterns of nicotine pouch usage among NP users

Among NP Users (*n* = 560), duration of NP use was most commonly 1–2 years (204, 36.4%), followed by <6 months (154, 27.5%) and 6 months to 1 year (153, 27.3%). Nearly half used 4–9 pouches per day (262, 46.8%), and 188 (33.6%) used 10–15 per day. Time to first NP after waking was most frequently 6–30 min (193, 34.5%) or within 5 min (170, 30.4%). Preferred NP strength was largely 10 mg (405, 72.3%). Most participants reported no change in nicotine strength since starting (288, 51.4%), while 94 (16.8%) reported gradually increasing strength and 74 (13.2%) started high and gradually tapered down. Concurrent use of multiple NP was commonly reported (rarely 197, 35.2%; sometimes 137, 24.5%). Mint was the most preferred flavor (374, 66.8%), and flavor was rated as the most important factor by 314 (56.1%). Most NP users reported using NP when ill (318, 56.8%). Table 2 presents patterns of NP use among NP users, including 95% confidence intervals for descriptive estimates.

Baseline cigarette dependence (FTND score) did not significantly predict preferred NP strength (χ^2^ = 7.544, df = 8, *p* = 0.479). However, FTND score was significantly correlated with pouches per day (Spearman rho = 0.346, *p* < 0.001), indicating that individuals with higher prior cigarette dependence used more pouches daily rather than selecting higher-strength products. The mean adapted FTND-ST score among NP Users was 5.89 ± 2.47 (median: 6.0; range: 0–12; *n* = 551; 9 NP Users had incomplete item responses). The adapted instrument scored six items: time to first pouch after waking (0–3), difficulty refraining in restricted places (0–1), which use period would be most hated to give up (0–1), pouches per day (0–3), use of multiple pouches at once (0–3), and use when ill (0–1), yielding a possible total score of 0–12. Given that two of six items were modified from the original FTND-ST and the response scale for the multiple-pouch item was expanded from binary to four-level, this score should be interpreted as a behavioral use pattern descriptor rather than a validated measure of nicotine dependence, and direct numerical comparison with the original FTND-ST total score is not recommended.

### User-reported perceptions: NP use relative to prior cigarette smoking

**Figure 2:**
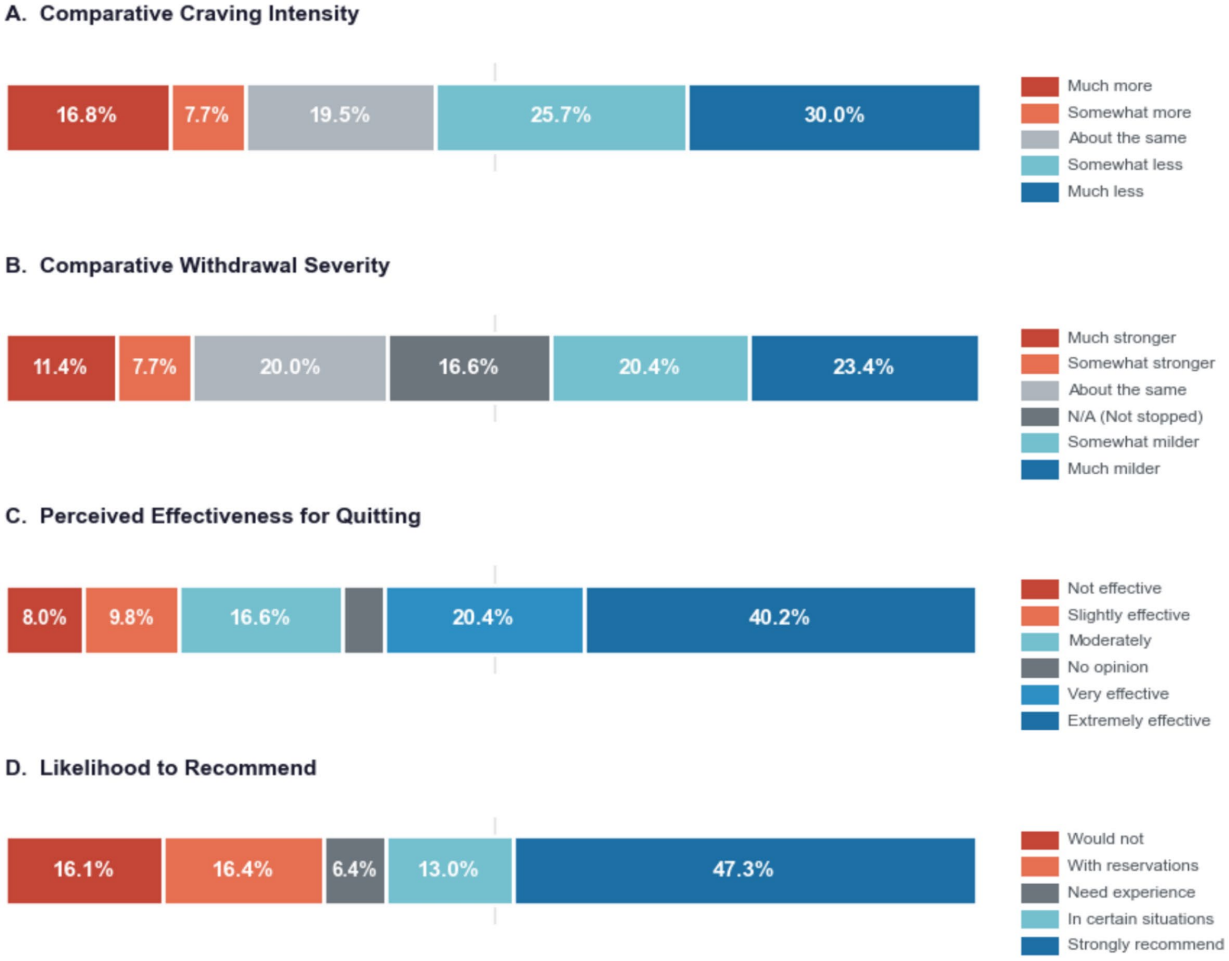
User-reported comparison of NP with combustible cigarettes. Diverging stacked bar chart illustrating user-reported outcomes across four domains (*N* = 560). **(A)** Comparative craving intensity: participants rated craving intensity during current pouch use relative to prior smoking. **(B)** Withdrawal severity: comparison of withdrawal symptom severity; “N/A” denotes participants who had not stopped using the product long enough to assess withdrawal. **(C)** Perceived effectiveness: subjective rating of NP as a smoking-cessation aid. **(D)** Likelihood to recommend: willingness to recommend NP to other smokers. Data are presented as percentages.

NP Users reported a favorable symptom profile during NP use relative to their recalled cigarette smoking experience. The majority of NP users reported that their craving intensity during NP use was lower than they recalled during cigarette smoking (55.7%), and 43.8% experienced milder withdrawal symptoms compared to smoking. Only 24.5 and 19.1% reported increased craving or withdrawal, respectively. Over 60% of the cohort rated NP as a “Very” or “Extremely” effective tool for smoking cessation. Furthermore, 47.3% of NP Users expressed willingness to recommend NP to peers, while only 16.1% stated they would not recommend them. Figure 2 illustrates comparison of NP and regular cigarette on craving intensity, withdrawal severity, perceived effectiveness for quitting, and likelihood to recommend NP for others.

### Multivariable predictors of consumption intensity

**Figure 3:**
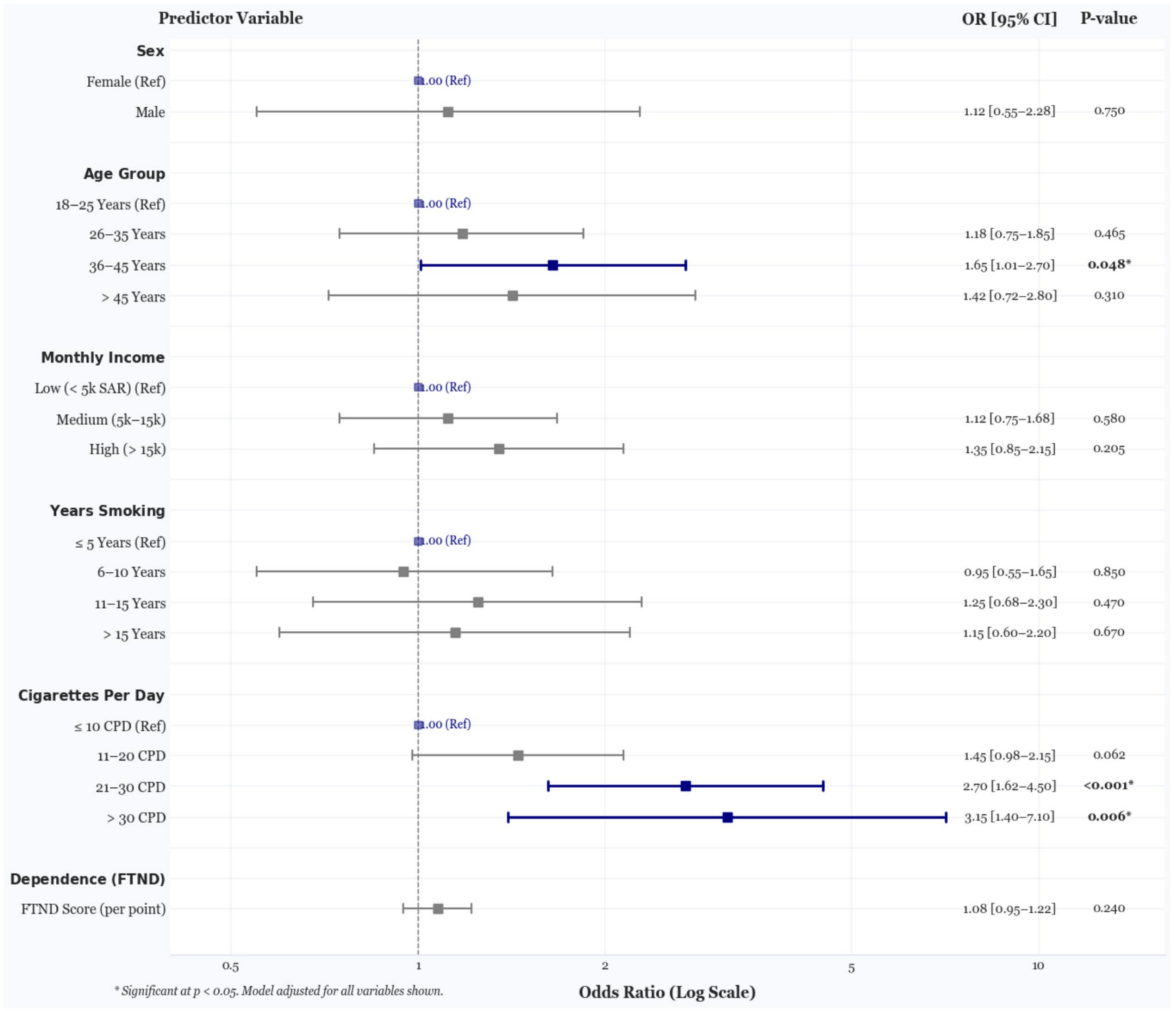
Multivariable predictors of high-intensity nicotine pouch consumption. Forest plot displaying adjusted odds ratios (aORs) and 95% confidence intervals from a multivariable logistic regression model predicting high-intensity nicotine pouch use (≥10 pouches/day). The model adjusted for sex, age group, monthly income, years of smoking, cigarettes per day, and Fagerström Test for Nicotine Dependence score. OR, odds ratio; CI, confidence interval; CPD, cigarettes per day; FTND, Fagerström Test for Nicotine Dependence. For the multivariable logistic regression, age groups 56–65 and >65 were collapsed into ≥56 years because of small cell counts.

The multivariable logistic regression model (*n* = 551; 9 NP Users excluded due to missing data) showed higher odds of high-intensity NP use (≥10 pouches/day) among participants with higher cigarette consumption. Compared with ≤10 CPD, those reporting 21–30 CPD had increased odds of high-intensity NP use (OR = 2.70; 95% CI: 1.62–4.50; *p* < 0.001), and those reporting >30 CPD had similarly increased odds (OR = 3.15; 95% CI: 1.40–7.10; *p* = 0.006). Participants aged 36–45 years also had increased odds compared with ages 18–25 (OR = 1.65; 95% CI: 1.01–2.70; *p* = 0.048). Baseline FTND score was not independently associated with high-intensity NP use (OR = 1.08 per point; 95% CI: 0.95–1.22; *p* = 0.240). Figure 3 presents multivariable predictors of high intensity NP consumption.

## Discussion

The findings provide evidence of NP use patterns among adults with a history of cigarette smoking. Most NP users preferred 10-mg products (405/560, 72.3%). A substantial proportion reported high-intensity NP use (≥10 pouches/day: 236/560, 42.1%). Approximately one in six participants (94/560, 16.8%) reported gradually increasing their nicotine strength over time, and an additional 17% reported fluctuating or switching between strengths as needed. Mint was the most preferred flavor among two-thirds of participants, and more than half of the participants reported that flavor plays an important role in their NP use. The present findings also indicate substantial NP craving; 363/560 (64.8%) used their first nicotine pouch within 30 min of waking.

NP users consumed relatively high nicotine doses at frequent intervals throughout the day. These findings are consistent with previous data reported among young medical students in Saudi Arabia (23). Alghamdi et al. found that 59% of participants reported 10 mg as their usual daily NP strength. A significant association was reported between craving control and NP of 10 mg (24). The Saudi NP market comprises both imported and locally manufactured products; however, locally manufactured products appear to be predominant, with a maximum available nicotine strength of 10 mg. Similarly, East et al. reported that NP strengths of 11–20 mg were the most commonly used among their surveyed sample (25). Nicotine strengths of 11–15 mg have also been reported among Swedish and Danish populations (26). In the present investigation, NP-related dependence could not be accurately assessed because a validated tool was not available. Nevertheless, the observed use patterns are consistent with behaviors commonly associated with nicotine dependence, though direct assessment was not possible with the available instruments. Dowd et al. used the FTND-ST to assess nicotine dependence among NP users. The authors reported evidence of significant nicotine dependence (21). Nicotine is a well-known addictive substance, which commonly leads to long-term use. In fact, most NP users adopt these products as a perceived safer alternative or as a tool to support smoking cessation (22). However, the use of high nicotine doses—as observed in our data and prior studies—and occasional dose escalation may reflect patterns commonly associated with nicotine dependence, although the level of NP-specific dependence could not be directly quantified. These findings raise questions about whether NP function primarily as a smoking cessation aid or as an alternative source of nicotine that may sustain long-term nicotine use.

Flavor was reported as the most important factor in NP product selection among NP users, with mint being the most preferred flavor. A Dutch survey reported that NP use in the general population was very low; however, flavors were identified as a key factor motivating initiation (27). Dowd et al. confirmed that flavor (mint and tobacco) was considered a motive to use (21). Similarly, an Australian survey of more than 1,500 participants reported that flavor was one of the two main motives for NP use, with fruit and menthol/mint being the most preferred flavors (22). Manufacturers increasingly use flavorings in both tobacco products and NP to enhance product appeal. This marketing approach may attract new users and encourage continued use, thereby potentially sustaining nicotine consumption (12). Flavorings enhance product appeal and enjoyment and may influence user experience, with some users reporting perceived reductions in stress or tension. Moreover, the aroma of flavored tobacco products enhances their appeal and attractiveness (28). Therefore, it is unsurprising that NP manufacturers use flavors to shape consumer preferences and potentially promote initiation and continued use. In fact, the effect of flavoring on nicotine uptake remains uncertain, and current pharmacokinetic evidence indicates minimal or no differences across flavors (29, 30). However, the authors reported that flavored varieties were associated with higher satisfaction, increased product attractiveness, and greater intention to reuse (29). While the present data demonstrate that flavor plays an important role in product selection, the survey did not directly assess reasons for continued NP use after initiation. Future studies should distinguish between factors influencing product choice and those driving sustained use.

Individuals who experience craving in both situations (cigarette smoking and NP use) are best positioned to compare the relative intensity of craving. The present data showed that NP-related craving intensity was lower than that of regular cigarettes, as reported by more than half of participants. This pattern is broadly consistent with evidence from the electronic nicotine delivery systems literature showing that, when smokers use alternative nicotine-delivery products with nicotine delivery sufficient to suppress smoking, dependence on the alternative product may be lower than on combustible cigarettes (31). However, more than 40% reported either similar or higher craving intensity when using NP. Przulj et al. found that NP deliver nicotine more slowly but may reach higher plasma concentration levels. Cigarettes reduced urges faster initially; however, this difference attenuated after 30 min, with similar urge reduction thereafter. Moreover, the effect of NP on reducing smoking urges appears to be more sustained than that of regular cigarettes (30). Fucito et al. reported that NP relieve nicotine urges to some extent; however, this effect may not be comparable to the craving relief achieved by regular cigarettes (15). This may partly explain why some users perceive NP as less satisfying than cigarettes. While our sample comprises complete switchers, recent evidence from electronic nicotine products suggests that dual use decomposes into two distinct modes: infrequent alternative product use with continued high-rate smoking, and frequent alternative product use with reduced smoking (32). Future studies should examine whether similar patterns exist among NP users who have not fully ceased cigarette smoking. Therefore, these findings collectively suggest that craving intensity differs between cigarettes and NP, with lower craving intensity reported for NP. Similar to craving intensity, the severity of withdrawal symptoms divided participants into two groups. Nearly 40% of participants reported that withdrawal symptoms associated with NP use were either comparable to those experienced with regular cigarettes or more intense, whereas 44% reported that withdrawal symptoms were less intense. This variability may be partly explained by individual differences in nicotine metabolism, which influence nicotine exposure (33, 34). This process may contribute to variation in nicotine dependence and withdrawal experiences (35). Thus, lower craving intensity and reduced withdrawal symptom severity may explain why 60% of NP users perceived NP as very or extremely effective for cessation; however, this reflects a subjective user perception, not a measured cessation outcome. It has been reported that craving intensity and withdrawal symptom severity are key determinants of continued smoking (35). Additionally, 47% of participants reported that they would recommend NP to other smokers as a smoking cessation aid. Similar perceptions have been reported previously, with participants viewing NP as a favorable cessation/harm-reduction approach, which may explain their willingness to recommend them to other smokers (24).

The present investigation showed that participants who reported higher prior cigarette consumption as indicated by CPD (>20) had higher odds of heavy NP use. Similarly, participants aged 36–45 years had higher odds of NP use compared with those aged 18–25 years. East et al. identified current smoking/vaping status and younger age as key correlates of NP use (25). Likewise, Jongenelis et al. reported that NP use was more common among current smokers/vapers and males (22). However, both studies did not investigate determinants of heavy NP use. Collectively, these findings suggest that NP are more likely to be used by individuals with established nicotine use patterns, potentially reflecting underlying nicotine dependence. Our findings suggest that individuals with higher prior cigarette consumption may use NP at higher frequency to sustain their usual nicotine intake. Indeed, FTND score was correlated with NP pouches per day (rho = 0.346, *p* < 0.001), but not with preferred NP strength (*p* = 0.479), suggesting that heavier smokers compensate through frequency rather than concentration. This dose-frequency relationship is consistent with Al-Nafisi et al., who reported positive associations between NP usage frequency and symptom prevalence among Saudi users (36). However, FTND score was not independently associated with high-intensity pouch use. Similarly, we did not find any statistically significant association between gender, monthly income, and years of smoking with the intensity of NP use. The lack of significant association between years of smoking and nicotine dependence suggests that higher dependence is probably related to the intensity of use rather than duration of use.

The present findings should be interpreted in the context of the Saudi Arabian NP market, which comprises both imported and locally manufactured products with a maximum nicotine strength of 30 mg. The high prevalence of 10-mg product use (72.3%) likely reflects market availability rather than user preference alone. This is consistent with Aldali, who reported that the locally manufactured brand DZRT accounted for 97% of products used by Saudi NP users, with a typical dosage of 7–10 mg (37). Because regulatory approaches to nicotine pouches vary substantially across countries, the generalizability of findings across settings may be limited (38).

**Figure 4:**
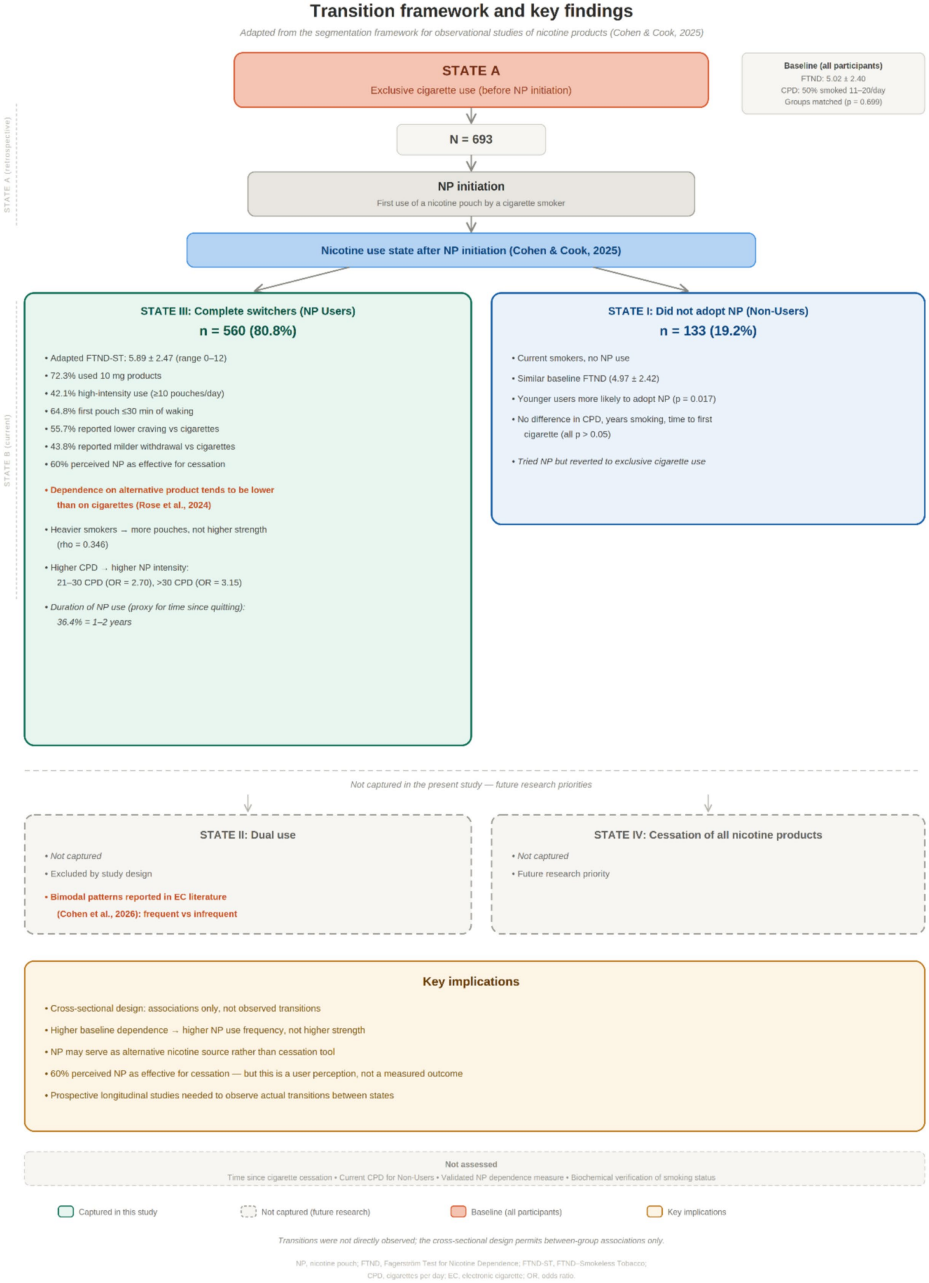
Conceptual framework of nicotine pouch transition states and key study findings (*N* = 693). Solid borders indicate states captured in the present study (States I and III); dashed borders indicate states not captured (States II and IV). Key findings for each captured state are summarized. Items not assessed include time since cigarette cessation, current CPD for Non-Users, a validated NP dependence measure, and biochemical verification of smoking status. Transitions were not directly observed; the cross-sectional design permits between-group associations only. NP, nicotine pouch; FTND, Fagerström Test for Nicotine Dependence; FTND-ST, FTND—Smokeless Tobacco; CPD, cigarettes per day; EC, electronic cigarette; OR, odds ratio.

Figure 4 presents the conceptual framework and key findings of the present study within the four-state transition model. All participants began in State A (exclusive cigarette use before NP initiation, defined as the first use of a nicotine pouch by a cigarette smoker). Adapted from the segmentation framework for observational studies of nicotine products (20), cigarette smokers who initiate NP use may enter one of four states: trial without adoption (State I), concurrent dual use (State II), complete switching (State III), or cessation of all nicotine products (State IV). The present study captured States I (Non-Users, *n* = 133) and III (NP Users, *n* = 560); States II and IV were not captured and represent priorities for future research. Evidence that dependence on alternative nicotine products tends to be lower than on combustible cigarettes (31) and that dual use decomposes into two distinct patterns of frequent and infrequent alternative product use (32) are noted as context from the electronic cigarette literature.

The present study provides valuable insight into NP use behaviors among adults with a history of cigarette smoking. However, several limitations should be acknowledged. First, the cross-sectional design cannot establish temporal relationships, assess behavioral transitions between nicotine products, or measure changes in dependence over time. All comparisons between cigarette smoking and NP use are based on retrospective self-report and should be interpreted as user perceptions, not objective assessments of product differences. Second, the study is subject to recall bias (retrospective FTND), selection bias (social media recruitment may overrepresent engaged NP users), and social desirability bias. The convenience sampling approach through online platforms limits the representativeness of the sample. Third, the lack of a validated instrument to assess NP-specific dependence limits the interpretability of dependence-related findings. The adapted instrument used in this study modified two of six FTND-ST items, which alters the underlying constructs measured. Nevertheless, the present study was able to identify the sociodemographic characteristics of NP users, explore their patterns and behaviors of use, and compare these findings with their previous smoking behaviors.

Fourth, the study recruited only complete switchers (NP Users) and current smokers (Non-Users). Dual users, individuals who use both cigarettes and NP concurrently, were not represented in the sample. Future studies should include dual users and individuals who have achieved complete nicotine cessation to capture the full spectrum of transitions. Fifth, women comprised only 3.8% of the sample, reflecting the low prevalence of tobacco and NP use among Saudi women and potential underreporting due to social norms. This gender distribution is consistent with other Saudi NP studies, where males comprised 63–92% of users (4, 24).

In summary, the present findings suggest that adults with a history of cigarette smoking report NP use patterns that appear broadly similar to their prior smoking behaviors. User-reported craving intensity and withdrawal symptoms were perceived as comparable to or lower than those recalled during cigarette smoking. NP may represent an alternative route of nicotine delivery. Prospective longitudinal studies and randomized controlled trials are needed to evaluate their effectiveness as a smoking cessation tool, to assess whether dependence is maintained, reduced, or eliminated during product transitions, and to characterize transitions between cigarette and NP use over time.

## Data Availability

The original contributions presented in the study are included in the article/Supplementary material, further inquiries can be directed to the corresponding author.
