# Supplementary material for: Patterns and characteristics of nicotine pouch use among adults with a history of cigarette smoking
Source: Front Public Health. 2026 Jun 15;14:1806892. doi: 10.3389/fpubh.2026.1806892 (PMC13310886; doi:10.3389/fpubh.2026.1806892)
Supplement: Supplementary file 1 [file Data_Sheet_1.PDF]

Comparison Between Cigarette Smoking and Nicotine Pouch Use

This survey is intended for individuals who were cigarette smokers and switched to using nicotine pouches. The aim is to understand how smokers' behavior changed before and after using nicotine pouches. Completing this survey will take no more than 3 minutes, and we kindly ask you to answer all questions. Thank you for your cooperation.

By selecting **(Yes)**, you confirm your consent to participate in this survey, with our full commitment to the confidentiality of your responses.

- **Yes, I agree to participate**

Gender:

• Male
• Female

Age:

• 18–25
• 26–35
• 36–45
• 46–55
• 56–65
• More than 65

Marital Status:

• Single
• Married

Education Level:

• High school or less
• Post-secondary diploma

• Bachelor’s degree
• Postgraduate studies (Master’s or PhD)

Monthly Income (Saudi Riyals):

• Less than 5000
• 5001–10000
• 10001–15000
• 15001–20000
• More than 20001

How old were you when you started smoking cigarettes?

• Less than 15 years
• 15–20 years
• 21–25 years
• 26–30 years
• 31–35 years
• More than 35 years

How many years did you smoke regular cigarettes?

• 5 years or less
• 6–10 years
• 11–15 years
• More than 15 years

How soon after waking up do you smoke your first cigarette?

• Within 5 minutes
• 6–30 minutes
• 31–60 minutes
• After 60 minutes

Did you find it difficult to refrain from smoking in public places where it is forbidden ?

• Yes
• No

Which cigarette would hate most to give up?

• The first cigarette in the morning
• Other cigarettes

How many cigarettes did you smoke per day?

• 10 or fewer
• 11–20
• 21–30
• 31 or more

Did you smoke more frequently during the first hours after waking compared to the rest of the day?

• Yes
• No

Did you smoke even when you are so ill that you are in bed most of the day?

• Yes
• No

Nicotine Pouch Use

How long have you been regularly using nicotine pouches?

• Less than 6 months
• 6 months to 1 year
• 1–2 years
• 2–3 years
• More than 3 years

What nicotine strength do you usually prefer when using nicotine pouches?

- 3 mg
- 6 mg
- 10 mg
- 15 mg
- 20 mg
- Other (please specify)

How has the nicotine strength you use changed since you started using nicotine pouches?

- I started with a low strength and gradually increased it
- I started with a high strength and gradually decreased it
- I have not changed the strength since the beginning
- I switch between different strengths as needed
- I changed the strength several times up and down

How important is flavor when choosing a nicotine pouch?

- Very important (the most important factor in my choice)
- Important (an important factor but not the most important)
- Moderately important
- Slightly important
- Not important at all

Which flavor do you prefer most in nicotine pouches? (Choose one)

- Mint
- Citrus fruits (lemon, orange)
- Berries (strawberry, blueberry)
- Tropical fruits (mango, pineapple)
- Coffee / caffeine
- Classic tobacco
- Sweet flavors (vanilla, caramel)
- Herbal / natural flavors
- Unflavored
- Other (please specify)

How soon after waking do you use your first nicotine pouch?

• Within 5 minutes
• 6–30 minutes
• 31–60 minutes
• After more than 60 minutes

Do you find it difficult to refrain from using nicotine pouches in places where their use is not allowed (e.g., workplace or public places)?

• Yes
• No

Which nicotine pouch is the most important to you or the one you cannot do without?

• The first pouch after waking up
• Any other pouch during the day

How many nicotine pouches do you usually use per day?

• 1–3
• 4–9
• 10–15
• More than 15

Do you use nicotine pouches when you are very ill to the point that you cannot perform your daily activities?

• Yes
• No

Have you ever used more than one nicotine pouch at the same time to increase nicotine strength?

• Yes, regularly
• Yes, sometimes
• Yes, only a few times
• No, never

Based on your personal experience, how would you compare the intensity of craving for nicotine pouches with the craving for cigarette smoking?

- Much less (nicotine pouches cause much less craving)
- Somewhat less
- About the same
- Somewhat more
- Much more (nicotine pouches cause much more craving than cigarettes)

When you stop using for a period of time, how do the withdrawal symptoms (such as stress, anxiety, difficulty concentrating, irritability) from nicotine pouches compare with those you experienced when stopping cigarettes smoking?

- Much less (withdrawal symptoms are much milder)
- Somewhat less
- About the same
- Somewhat more
- Much more (withdrawal symptoms are much more severe)
- I have not stopped using nicotine pouches long enough to judge

To what extent do you believe nicotine pouches are effective as a smoking cessation aid?

- Very effective (better than most other methods)
- Highly effective
- Moderately effective
- Slightly effective
- Not effective at all
- I do not have a specific opinion

To what degree would you recommend nicotine pouches as an alternative to smoking?

- I strongly recommend them for any smoker who wants to quit
- I recommend them for smokers under certain conditions
- I might recommend them with some reservations
- I do not recommend them at all
- I need more experience before recommending them
